# Beyond new vaccine introduction: the uptake of pneumococcal conjugate vaccine in the African Region

**DOI:** 10.11604/pamj.supp.2017.27.3.11531

**Published:** 2017-06-21

**Authors:** Folake Olayinka, Leah Ewald, Robert Steinglass

**Affiliations:** 1 USAID’s Maternal and Child Survival Program/John Snow, Inc, USA

**Keywords:** Immunization, vaccination, pneumonia, child mortality, Africa

## Abstract

The number of vaccines available to low-income countries has increased dramatically over the last decade. Overall infant immunization coverage in the WHO African region has stagnated in the past few years while countries’ ability to maintain high immunization coverage rates following introduction of new vaccines has been uneven. This case study examines post-introduction coverage among African countries that introduced PCV between 2008 and 2013 and the factors affecting Pneumococcal Conjugate Vaccine (PCV) introduction. Nearly one-third of countries did not achieve 80% infant PCV3 coverage by two years post-introduction and 58% of countries experienced a decline in coverage between post introduction years two and four. Major factors affecting coverage rates included introduction without adequate preparation, insufficient supply chain capacity and management, poor communication between organizations and with the public, and data collection systems that were insufficient to meet information needs. Deliberately addressing these issues as well as longstanding weaknesses during new vaccine introduction can strengthen the immunization and broader health system. Further study is required to identify and address factors that affect maintenance of high coverage following introduction of new vaccines in the African region. Immunization with PCV is one of the most important interventions protecting against pneumonia, the second leading cause of death for children under five globally.

## Introduction

The last ten years have seen a massive surge in vaccine products and many African children and adults have gained access to life-saving immunization against deadly diseases. A global increase in innovative financing and advances in the production of new vaccines have allowed low-income countries to introduce new vaccines faster than ever before. Between 2000 and 2015, 73 Gavi eligible countries introduced a pentavalent vaccine, 54 introduced pneumococcal conjugate vaccine, 37 introduced rotavirus vaccine, 40 introduced inactivated polio vaccine, 20 introduced measles vaccine second dose, two introduced HPV vaccine nationally and one introduced Japanese encephalitis vaccine [1]. Pneumonia remains the second biggest killer of children under five years of age and is responsible for one-sixth of child deaths in the African region [2], and disproportionately affects children from poor and disadvantaged households [3]. Pneumonia deaths are largely preventable with effective interventions such as vaccination against whooping cough (pertussis), measles, Haemophilus influenzae type b (Hib) and pneumococcus [4]. Ensuring exclusive breastfeeding for six months and continued breastfeeding complemented by nutritious solid foods up to age two, providing safe drinking water and sanitation [5], and reducing indoor pollution [6] also protects children from respiratory infection. Despite the large number of countries introducing the pneumococcus conjugate vaccine (PCV) in recent years, the latest report from Gavi, the Vaccine Alliance, notes that coverage rates remain low in Gavi eligible countries [1]. This case study examines the uptake of the third dose of pneumococcal conjugate vaccine (PCV3) introduced to countries in the African region between 2008 and 2014. Consideration is given to factors that affect sustained vaccination and delivery following introduction. The case study objectives are to identify the enablers of uptake of PCV3 into country routine immunization systems and highlight the lessons learned in the process.

## Methods

In conducting this case study, we collected information on dates of nation-wide introduction as reported in Gavi annual progress reports. We examined PCV 3 coverage data from the WHO/UNICEF Joint Reporting Form [7] and used it to categorize countries as having high infant coverage (over 80%), moderate coverage (50-80%) or low coverage (below 50%) at one, two and four years post-introduction. For example, in defining years post-introduction for a vaccine introduced in 2012, we considered one year post-introduction coverage to be that recorded by the JRF in 2013, and two-year post-introduction coverage to be that reported by the JRF in 2014. We then reviewed information about enabling and inhibiting factors for high coverage rates from country post introduction evaluations, Gavi reports and the USAID-funded Maternal and Child Health Integrated Program (MCHIP) report [8].

## Case Study

**Figure 1:**
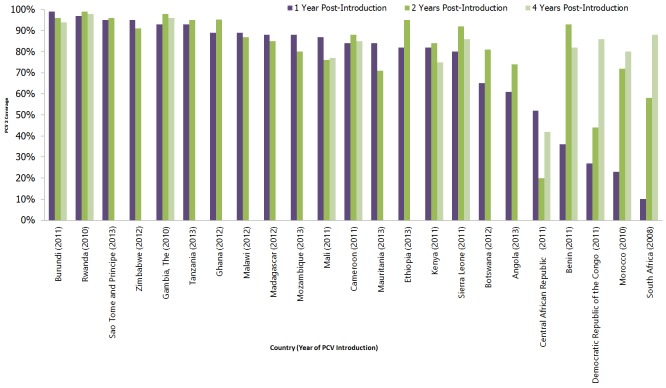
PCV3 coverage in African countries introducing PCV from 2008 to 2013, data from WHO/UNICEF Joint Reporting Form

PCV had been introduced in 129 countries worldwide by the end of 2015. PCV was introduced in 22 of 45 countries in the WHO African Region between 2008 and 2013 (Figure 1) [7]. However, of the 15 countries that introduced PCV before 2014 and achieved greater than 80% coverage by first year post-introduction (Table 1); eight maintained or increased their coverage by post-introduction year two, while seven countries’ coverage declined. Mali and Mauritania fell below the 80% threshold. Out of the 11 countries that introduced the vaccine before 2012, seven experienced a decline in PCV3 coverage in post-introduction years two through four, while PCV 3 coverage dropped below 80% in Kenya. The Central African Republic achieved 52% in post-introduction year one, but had dropped to 42% by post-introduction year four. Only eight countries (Burundi, Rwanda, the Gambia, Cameroon, Sierra Leone, Benin, DRC, and South Africa) had achieved or maintained over 80% coverage at four years post-introduction. Both South Africa and DRC improved coverage from below 50% in post introduction year one to over 80% in post-introduction year four. Given that the introduction of new vaccines provides an opportunity to equitably immunize more populations against diseases, the ability to continue to do so beyond the first few years is critical.

**Table 1:** PCV3 and DTP3 coverage one, two and four years post PCV introduction in African countries that achieved pcv3 coverage above 80% in the first year

	1 Year Post-Introduction		2 Years Post-Introduction		4 Years Post-Introduction	
Country (Year of PCV Introduction)	PCV3 Coverage	DTP3 Coverage	PCV3 Coverage	DTP3 Coverage	PCV3Coverage	DTP3Coverage
Burundi (2011)	99%	96%	96%	96%	94%	94%
Rwanda (2010)	97%	97%	99%	99%	98%	98%
Sao Tome and Principe (2013)	95%	95%	96%	96%		
Burkina Faso (2014)	91%	91%				
Zimbabwe (2012)	95%	95%	91%	91%		
Gambia, The (2010)	93%	96%	98%	98%	96%	96%
Tanzania (2013)	93%	97%	95%	98%		
Cote d’Ivoire (2014)	81%	91%				
Ghana (2012)	89%	90%	98%	98%		
Malawi (2012)	89%	89%	87%	91%		
Madagascar (2012)	88%	90%	85%	89%		
Mozambique (2013)	88%	88%	80%	80%		
Mali (2011)	87%	90%	76%	76%	77%	90%
Cameroon (2011)	84%	85%	88%	89%	85%	87%
Mauritania (2013)	84%	84%	71%	73%		
Ethiopia (2013)	82%	84%	95%	96%		
Kenya (2011)	82%	83%	84%	84%	75%	78%

Data from WHO/UNICEF Joint Reporting Form [7]

Despite high coverage within the immediate introduction period, more than half of the countries experienced some decline by post-introduction year two. Some further decline was seen in some countries two to four years after introduction of the vaccines. Comparing PCV rates with those of diphtheria, pertussis and tetanus (DPT) vaccine, which is given at the same age, in countries with over 80% one year post-introduction, PCV 3 was at similar levels of coverage as the third dose of DPT within two years for most countries. However, in Mauritania, Cameroon, Madagascar, Ethiopia, Tanzania and Malawi, PCV3 coverage remained slightly below that of DPT 3. Further comparison in post-introduction year four shows that Mali, Cameroon and Kenya reported lower PCV3 coverage rates than DPT3, with Mali reporting as much as 13 percentage points less. Factors that enabled high coverage included robust planning and forecasting with sufficient stock levels, strong partnership and coordination amongst government agencies and partners, and adequate and timely disbursement of funds.

Oversight by country Interagency Coordinating Committees (ICCs) and partnership between the Ministry of Health and key stakeholders, strengthened by regular meetings and good communication from the National Immunization Program, were major enablers in a number of countries such as Mozambique, Zimbabwe, Uganda and Zambia [9]. A phased introduction in DRC and Rwanda promoted a strong roll-out and also provided opportunity to learn lessons and adjust where necessary [8]. Rwanda’s success in introducing PCV and four other vaccines with coverage rates of over 90% has also been attributed to beginning a cross-sectoral planning process a full year before introduction; a careful review of disease burden data; the engagement of local leaders, development partners, civil society organizations, and health workers in communication efforts; the use of community health workers to confirm the target population size ahead of time; the promotion of government ownership; and the integration of vaccine introduction with other community-based interventions [10].

Zimbabwe avoided national stock out in the first year by ordering the second quarter´s shipment early and requesting additional vaccine from Gavi, while Malawi also received large initial stock amounts [8, 11]. Following the post-introduction evaluation, Zimbabwe also increased cold chain capacity and established contingency plans in case of stock-out [11]. In Tanzania, eligibility criteria stated that only children in the new birth cohort could receive the vaccine and no catch-up doses would be administered. Health workers were carefully informed that this policy would avoid stock outs. Additionally, parents received informational materials explaining the age group most at risk [8]. Some factors that inhibited countries’ ability to achieve high uptake of PCV3 included challenges ranging from stock availability, health worker capacity, regular and sufficient funding, management at subnational levels and coordination. For example, in Uganda (which introduced PCV in 2014 and is therefore not included in the table), changes in leadership and the introduction of a new financial system led to poor planning and insufficient disbursement of funds. In some instances, these funding challenges led to a scaling back of the introduction without adjustment in the social mobilization plans. As a result, caregivers brought their children for the vaccines only to discover PCV had not been introduced in their district and their children could not be vaccinated [9]. Eligibility guidelines provided a further challenge: even though the guidelines allowed for children over 12 months old to receive one catch-up dose, health workers were not instructed to provide it to this age group during training [8]. Additionally, the training of health workers was complicated by the lack of vaccine availability early on, which made practical demonstrations impossible. As such, trained health workers could not train other health workers as expected. In Rwanda, adjustments due to difficulty with waste management of pre-filled glass syringes resulted in switching from PCV 7 to PCV 13 in 2011 [12]. DRC also reported infrastructural challenges with waste management [13]. Some countries such as Kenya and Tanzania had to procure a different formulation of PCV than their preference due to global vaccine shortages of PCV 13 [8]. Furthermore, in a number of countries, challenges encountered in the course of introduction of new vaccines included stock-outs due to unclear eligibility policies (e.g., whether infants who had previously begun their pentavalent vaccination series and children more than 12 months old were eligible for any or all of the PCV series), poor vaccine management and insufficient funding for transport and fuel for cold chain equipment, inadequate systems to manage the increasing volume of waste generated by each new vaccine, health management information system (HMIS) forms that were not updated in a timely fashion, weak or nonexistent surveillance for adverse events following immunization (AEFI), and insufficient numbers of health workers trained on the new vaccine [8].

## Discussion

Largely through the support of Gavi, low-income countries in Africa now have greater access to new vaccines impacting major causes of death. Overall child mortality fell by over 53% in Africa between 1990 and 2012 [14]. Between 2000 and 2015, the number of deaths of children under five fell by 85% for measles; 59% for pertussis, tetanus and meningitis; 58% for malaria; 57% for diarrhea and 47% for pneumonia [2]. Diseases caused by Streptococcus pneumonia (S. pneumonia or pneumococcus) are major public health problems worldwide. Pneumococcal disease is caused by bacteria that can spread from person to person through close contact. In addition to ear infections and bronchitis, it can lead to more serious infections such as pneumonia, bacteremia and meningitis. PCV 13 protects against 13 types of pneumococcal bacteria. For PCV administration to infants, the WHO recommends three primary doses without boosters (the 3p+0 schedule) or two primary doses plus a booster (the 2p+1 schedule). In choosing between the 3p+0 and 2p+1 schedules, countries should consider locally relevant factors including the age distribution of pneumococcal disease, likely vaccination coverage, and timeliness of vaccination doses [15]. Drawing lessons from introduction of new vaccines more broadly, operational and programmatic readiness at national and subnational levels are critical factors for successful introduction of new vaccines. Yet, high-level political interest at the global and national level to launch new vaccines sometimes primarily focuses on development, supply, cost and finance of the vaccines. Planning and preparation for introduction of new vaccines can highlight weaknesses in the immunization programs, providing an opportunity to strengthen systems and address the deficiencies. Other factors that may play important roles include the effect of civil unrest, conflict and health system reforms. A frequent missed opportunity was not taking advantage of the high political attention which always accompanied new vaccine introduction to address chronic problems with long-term solutions.

New vaccine introductions posed particular challenges to timely procurement and supply chain management. Cold chain capacity was expanded in many countries with acquisition of equipment and devices; however, cold chain repair and continued preventive maintenance were often neglected. In some instances waste management was given inadequate attention with several challenges in the management and disposal of the increased volume of medical sharps waste generated by the additional injectable vaccines. New vaccines are generally accepted by the population and generate demand for immunization, but this increase in demand also highlights the importance of good communication with the public. Opportunity to build health worker capacity to address parental and health worker concerns about the new and unfamiliar vaccines was at times not utilized and the eligibility criteria was not always clearly explained. This resulted in mixed messages being communicated and, in some instances, stock-out of vaccines, particularly in the year of introduction. Finally, vaccine introduction programs must place an early emphasis on the updating of data collection and storage procedures in order to adequately monitor coverage and predict supply needs. Paper records and electronic information systems should be adjusted and made available before introduction and must be available as soon as rollout begins. Unfortunately, the development and strengthening of AEFI and surveillance systems has been inadequate in many countries, and continues to lag behind the new vaccines introduced. When detailed technical guidance and adequate funding is available, new vaccine introduction can present an opportunity to make system-wide improvements that address problems such as surveillance, training, cold chain and logistical capacity, and injection safety system-wide. However, it has been shown that these opportunities are frequently missed due to poor planning around financial and human resources needs [16]. In a couple of countries, PCV3 coverage trailed behind vaccines given at the same time such as DPT3 years after the introduction, warranting greater attention to particular country factors.

## Conclusion

The introduction of new vaccines such as PCV (13) not only provides a major opportunity to reduce childhood deaths from one of the major causes of mortality in children, but can also provide a platform to strengthen immunization and health systems, particularly in the areas of health worker capacity, service delivery, recording tools, information management, logistics and vaccine supply systems, communications, community engagement, coordination and partnership. Leveraging the political will to address chronic systemic issues related to low immunization rates and strengthen the broader health system remains a potential but often under-utilized opportunity. In future, regular reviews, updating of guidelines and application of the lessons learned through country experience in not only introducing, but sustaining high and equitable performance will go a long way towards increasing the uptake of new vaccines in the years ahead. Consistently, country experiences draw attention to the essential role of health systems in countries’ ability to achieve and sustain high coverage years following the introduction of new vaccines. Given that several introductions of new vaccines are planned in the coming years in the African region, more detailed study will be required to identify these factors and how they interrelate with sustained immunization uptake of new vaccines beyond the introductory period.

## Competing interests

The authors declare no competing interest.
